# *In Vitro* Selective Antibacterial and Antiproliferative Effects of Ethanolic Extracts from Cambodian and Philippine Plants Used in Folk Medicine for Diarrhea Treatment

**DOI:** 10.3389/fphar.2021.746808

**Published:** 2021-11-26

**Authors:** Tomas Kudera, Barbora Fiserova, Marie Korytakova, Ivo Doskocil, Hana Salmonova, Edgardo E. Tulin, Samnang Nguon, Marlito M. Bande, Ladislav Kokoska

**Affiliations:** ^1^ Laboratory of Ethnobotany and Ethnopharmacology, Department of Crop Sciences and Agroforestry, Faculty of Tropical AgriSciences, Czech University of Life Sciences Prague, Prague, Czechia; ^2^ Faculty of Agrobiology, Food and Natural Resources, Department of Microbiology, Nutrition and Dietetics, Czech University of Life Sciences Prague, Prague, Czechia; ^3^ PhilRootcrops, Visayas State University, Baybay, Philippines; ^4^ Graduate School, Royal University of Agriculture, Phnom Penh, Cambodia; ^5^ Institute of Tropical Ecology and Environmental Management, Visayas State University, Baybay, Philippines

**Keywords:** diarrhea, Cambodia, Philippines, medicinal plant, antibacterial, anticancer

## Abstract

Bacterial diarrhea remains a global health problem, especially in developing tropical countries. Moreover, dysbiosis caused by diarrheagenic bacteria and inappropriate antimicrobial treatment has been associated with intestinal carcinogenesis. Despite the rich tradition of the use of herbs for the treatment of gastrointestinal disorders in Cambodian and Philippine folk medicine, many of them have not yet been systematically studied for their *in vitro* selective inhibitory effects on intestinal bacteria and cells. In the present study, *in vitro* inhibitory activities of 35 ethanolic extracts derived from 32 Cambodian and Philippine medicinal plants were determined by broth microdilution method against 12 pathogenic bacteria. Furthermore, cytotoxicity against intestinal cancer cells (Caco-2 and HT-29) using thiazolyl blue tetrazolium bromide cytotoxicity assay and safety to six beneficial intestinal bacteria (bifidobacteria and lactobacilli) and intestinal normal cells (FHs 74 Int) were determined for the antimicrobially active extracts. Selectivity indices (SIs) were calculated among the averages of minimum inhibitory concentrations (MICs), half-maximal inhibitory concentrations (IC_50_), and 80% inhibitory concentrations of proliferation (IC_80_) for each type of the tested agents. The extracts of *Artocarpus blancoi* (Elmer) Merr. (Moraceae), *Ancistrocladus tectorius* (Lour.) Merr. (Ancistrocladaceae), and *Pentacme siamensis* (Miq.) Kurz (Dipterocarpaceae) produced significant growth-inhibitory effects (MICs = 32–512 μg/ml) against intestinal pathogenic bacteria at the concentrations nontoxic to normal intestinal cells (IC_80_ values >512 μg/ml; SIs = 0.11–0.2). Moreover, the extract of *P. siamensis* (Miq.) Kurz was relatively safe to beneficial bacteria (MICs ≥512 μg/ml; SI = 0.1), and together with *A. blancoi* (Elmer) Merr., they selectively inhibited intestinal cancer cells (IC_50_ values ≥51.98 ± 19.79 μg/ml; SIs = 0.3 and 0.6). Finally, a strong selective antiproliferative effect on cancer cells (IC_50_ values 37.89 ± 2.68 to 130.89 ± 13.99 μg/ml; SIs = 0.5) was exerted by *Ehretia microphylla* Lam. (Boraginaceae), *Lagerstroemia cochinchinensis* Pierre ex Gagnep. (Lythraceae), and *Melastoma saigonense* (Kuntze) Merr. (Melastomataceae) (leaves with flower buds). The results suggest that the above-mentioned species are promising materials for the development of new selective antibacterial and antiproliferative agents for the treatment of infectious diarrhea and associated intestinal cancer diseases. However, further research is needed regarding the isolation and identification of their active constituents.

## Introduction

According to the latest data of the World Health Organization, infectious diarrhea is still the third leading cause of death among all communicable diseases worldwide, especially affecting under-five children in developing countries (World Health Organization, 2020a). Moreover, common risk factors associated with these gastrointestinal infections, such as inappropriate changes in the host-gut microbiome (Sun et al., 2018), have been considered as a crucial precondition for several noncommunicable intestinal diseases, including colorectal cancer, which is the third leading cause of cancer death globally (Taddese et al., 2020). The developed countries are at the highest risk, but the incidence of gastrointestinal cancers in developing nations is steadily increasing (Rawla et al., 2019). Infectious and toxigenic strains of *Bacillus cereus*, *Campylobacter jejuni*, *Clostridium difficile*, *Clostridium perfringens*, *Enterococcus faecalis*, *Escherichia coli*, *Listeria monocytogenes*, *Salmonella* spp., *Shigella* spp., *Vibrio cholerae*, *Vibrio parahaemolyticus,* and *Yersinia enterocolitica* are the major causes of bacterial diarrhea (Casburn-Jones, 2004). Among these, *Enterococcus* spp., *Escherichia* spp., and *Shigella* spp. were previously observed to be richer in the fecal microbiota of patients with colorectal cancer (Xu and Jiang, 2017).

Despite the advantages of treatment utilizing antibiotic agents, disruption of the gut microbiota is usually considered as one of the negative consequences of their use in infectious diarrhea (Francino, 2016). Moreover, the antimicrobial resistance rate among diarrheagenic bacteria recovered from human patients has significantly increased, especially in developing countries (Meng et al., 2011; World Health Organization, 2020b). Therefore, it is important to seek new sources of efficient antimicrobial agents against which the infectious bacteria are less prone to develop resistance (Sibanda and Okoh, 2007). Additionally, agents that are highly selective, thus less disruptive for human microbial ecology, should be preferred (Garrett, 2019). Therefore, an investigation on the *in vitro* antimicrobial effects of promising candidates is recommended to involve both representatives of diarrheagenic and probiotic bacteria. The most common bacteria recognized to date as probiotics are *Lactobacillus* spp. and *Bifidobacterium* spp., belonging to the dominant bacterial phyla that can be found in human intestines (Behnsen et al., 2013). Although it is a simplified representation of beneficial gut microbiota, the counterscreen of *in vitro* inhibitory activities on gut commensals appears to be an effective way to avoid unnecessarily promiscuous agents (Gavrish et al., 2014).

Additionally, assessment of interactions with intestinal epithelial cells and toxicity profiles is another important factor for the evaluation of antibacterial agents, which target the intestinal site (Maher and McClean, 2006). To lower the potential toxic responses of intestinal epithelial cells, the use of antibacterial agents with mechanisms enabling toxicity to be prokaryotic but not eukaryotic cells, such as in the case of the antibiotic ceftriaxone, should be prioritized (Neftel and Hübscher, 1987). However, the antiproliferative activity can be acknowledged in cases where the immune responses caused by ongoing intestinal infection and dysbiosis have already promoted the carcinogenesis of epithelial cells. Initially, colorectal cancer usually has an oligosymptomatic characteristic; thus, many cases are diagnosed only at advanced stages, at which stage the therapeutic outcomes are poor (Rogowski and Sulzyc-Bielicka, 2016). Antibacterial agents able to eliminate diarrheagenic pathogens and having the additional selective antiproliferative properties could potentially help to prevent progression of yet not diagnosed intestinal cancers. The use of antibiotics—such as quinolones and tetracyclines, which are both utilized in diarrheal infections—as anticancer drugs has been previously suggested (Onoda et al., 2005; Batalha et al., 2016).

Plant-derived products provide novel chemical scaffolds for anti-infective drugs and leads that have chemically been modified and developed as antimicrobial agents. Several over-the-counter pharmaceuticals, dietary supplements, and herbal medicines recommended for the support and maintenance of gastrointestinal health, containing antibacterially active plant extracts and derivatives of their constituents, are already available at the international market. The benzylisoquinoline alkaloid berberine (e.g., *Hydrastis canadensis* L. [Ranunculaceae]); simple phenol bismuth subsalicylate, the analog of salicylic acid derived from salicin (*Salix alba* L. [Salicaceae]) (Kokoska et al., 2019); and picrosides, an iridoid glycoside of *Picrorhiza kurroa* Royle ex Benth. (Plantaginaceae), are some examples (Rathee et al., 2016). The *in vitro* selective antibacterial effects of plant-derived products have also been reported. For example, Chan et al. (2018) reported that the phenolic-rich extracts from various dietary spices and medicinal herbs (*Cinnamomum burmannii* Nees and T.Nees] Blume [Lauraceae], *Cinnamomum cassia* [L.] J.Presl [Lauraceae], *Origanum vulgare* L. [Lamiaceae], *Punica granatum* L. [Lythraceae], *Reynoutria japonica* Houtt. [Polygonaceae], and *Syzygium aromaticum* [L.]. Merr. and L.M.Perry [Myrtaceae]) exerted *in vitro* growth-inhibitory effects against selected foodborne pathogenic bacteria but not against lactic-acid bacteria. Selective *in vitro* antibacterial activity was also described in the study by Novakova et al. (2013), where the anticlostridial effect of 8-hydroxyquinoline (*Microstachys corniculata* [Vahl] Griseb. [Euphorbiaceae]) was higher than the activities revealed against different strains of bifidobacteria. In our previous study, we also reported that 8-hydroxyquinoline exerts selective *in vitro* antiproliferative activity against some intestinal cancer cell lines with a comparably lower effect on normal cells (Kudera et al., 2020). Quinoline alkaloids, such as camptothecin extracted from the bark of *Camptotheca acuminata* Decne. (Cornaceae), are already being used in chemotherapy for the treatment of colon cancer (Zeng et al., 2013). Anticancer activities of other antibacterially active phytochemicals (e.g., berberine) utilized against infectious diarrhea are currently studied (Lin et al., 2008). Based on these studies, it is evident that plant-derived products have great potential for the development of antibacterial and anticancer preparations for the treatment of infectious diarrhea and associated intestinal cancer diseases. Although many of such products work based on antidiarrheal activity (e.g., antisecretory and astringent effects) (Palombo, 2006), their antimicrobial effect is not an uncommon feature.

The Southeast Asian region is one of the world’s major sources of useful plant resources and has long been recognized as a center of plant biodiversity (Duriyaprapan et al., 2005). Situated in the humid tropics with areas of high rainfall, Southeast Asia has one of the largest numbers of vascular plants species globally. For centuries, people living in this region have relied on traditional medicine using available plants for daily healthcare. Cambodia and the Philippines are two geographically distinct Southeast Asian countries, each having numerous plant biodiversity hotspots and a long tradition of herbalism (de Padua et al., 1999). While the former is situated in the mainland, having rich ecosystems, especially around the Mekong River (Chassagne et al., 2016), the latter is a huge archipelago consisting of approximately 7,107 islands, many of which are the center of endemicity and biodiversity (Guzman et al., 2016).

Diarrhea has been a significant issue in both Cambodia and the Philippines (Our World in Data, 2011). Therefore, plant resources in these countries have extensively been utilized medicinally to treat this ailment. The Philippines also has the highest estimated number of cases of colorectal cancer in Southeast Asia and the tenth highest number of deaths in the world (Rawla et al., 2019). In certain provinces of Cambodia, treatment of digestive disorders, such as abdominal pain (chhu poh), diarrhea (reak ach), and dysentery (reak muol), has particularly been based on herbal medicine. Alcohol maceration is a common method of preparation of antidiarrheal medicines, whereas a majority of the preparations are administered orally: drunk, eaten, or chewed (Kham, 2004). Grilling the plant part over a fire and then boiling it into a form of decoction is also common. As an example, Bunong people in Mondulkiri province treat diarrhea using a “step-by-step” process using a sole ingredient from one plant that is substituted by a different species if the condition becomes persistent. In the Philippines, conditions such as diarrhea (pagtatae) and dysentery (pagdidisenyo) have similarly been treated by orally administered herbal preparations that are processed by alcohol maceration, decoction, or infusion or eaten and chewed raw. According to the Philippine traditional medicinal system, the disease is usually conceptualized as a disruption (dys-krasia) of the balance of forces (whether germs or evil spirits), both external and internal to humans. Therefore, it can be assumed that the use of herbal preparations is intended to also defend the immunological mechanisms, helping the body to overcome the disease itself (Tan, 1980). Despite the existence of several reports on the antibacterial and antiproliferative effects of Cambodian and Philippine medicinal plants used for the treatment of diarrhea (Beloy et al., 1976; Chea et al., 2007), there are several species in both regions that have not yet been appropriately studied using modern scientific techniques. In this study, we, therefore, examine the *in vitro* selective antibacterial and antiproliferative effects of ethanolic extracts from various parts of plant species that have been used in Cambodian and Philippine traditional herbal systems the treatment of gastrointestinal disorders and determine which bioactive properties have not been properly tested in such form and degree before.

## Materials and Methods

### Plant Materials

The criteria for selection of promising plant species included their uses for the treatment of diarrhea, dysentery, abdominal pain, and other gastrointestinal complaints in traditional herbal systems of Southeast Asia, particularly Cambodia and the Philippines. Therefore, the appropriate literature on ethnobotany and ethnomedicine of this region was primarily used (Chassagne et al., 2016; de Padua et al., 1999; van Duong, 1993; Kham, 2004; Langenberger et al., 2008; Lemmens and Bunyapraphatsara, 2003; Lim, 2012; Stuart, 2017; Tan, 1980; van Valkenburg and Bunyapraphatsara, 2001). Additionally, several species were identified through meetings with local herbalists in Cambodia (2) and the Philippines (1), assembled by local experts Dr. Nguon and Dr. Bande, respectively. Overall, more than 100 plant species were selected, referring to a limited number of previous studies testing their bioactivity *in vitro.* A total of 35 samples from different parts (bark, fruit, leaves, or roots, one per plant except three of the species) of 13 Cambodian and 19 Philippine medicinal plant species were collected from various locations in the Republic of the Philippines in April–May 2017 and 2018 and in the Kingdom of Cambodia in March–April 2019 (Table 1). The collected fresh samples were subsequently air-dried for several days and sent to the Czechia for further processing and bioactivity testing. Ethnobotany expert Prof. Kokoska and local experts Dr. Bande and Dr. Nguon authenticated the species. Their voucher specimens have been deposited in the herbarium of the Department of Botany and Plant Physiology of the Faculty of Agrobiology, Food and Natural Resources of the Czech University of Life Sciences Prague (Prague, Czechia). The scientific names of the collected species were reviewed using (The Plant List, 2013), and their local names were verified with data from literature and local herbalists (Tan, 1980; van Duong, 1993; de Padua et al., 1999; van Valkenburg and Bunyapraphatsara, 2001; Lemmens and Bunyapraphatsara, 2003; Kham, 2004; Langenberger et al., 2008; Lim, 2012; Chassagne et al., 2016; Stuart, 2017). For all assayed species, the scientific names, families, local names, voucher specimen codes, GPS coordinates, collected parts (plant samples), and their uses in folk medicine are presented in Table 1.

**TABLE 1:** Ethnobotanical data on Cambodian and Philippine medicinal plants.

Latin name (family)	GPS coordinates (country)	Local name	Voucher specimen	Tested part(s)	Extract yield (%)	Ethnomedicinal use
*Aganonerion polymorphum* Spire (Apocynaceae)	12.3966000N, 107.1934975E (C)	Vor Thneung	02559KBFRC	Whole plant	19.7	Diarrhea (Chassagne et al. (2016))
*Acalypha grandis* Benth. (Euphorbiaceae)	10.7623297N, 124.8062889E (P)	Unknown	02537KBFR8	Leaves	23.6	Diarrhea and dysentery; sapped/crushed into water/food (van Valkenburg and Bunyapraphatsara (2001))
*Acanthus ebracteatus* Vahl (Acanthaceae)	10.6888289N, 124.7956483E (P)	Diluario	02505KBFR3	Whole plant	17.9	Abdominal pain; decoction of 30–60 gDW (Stuart. (2017))
*Ancistrocladus tectorius* (Lour.) Merr. (Ancistrocladaceae)	13.7333775N, 107.0151108E (C)	Khan Maa	02560KBFR4	Leaves	16	Dysentery; decoction (Lemmens and Bunyapraphatsara (2003); interviewed herbalist)
*Aporosa villosa* (Lindl.) Baill. (Phyllanthaceae)	12.3965292N, 107.1938597E (C)	Krong	02561KBFR5	Leaves	6.1	Diarrhea and abdominal pain; decoction (Chassagne et al. (2016))
*Artocarpus blancoi* (Elmer) Merr. (Moraceae)	10.7435833N, 124.8020564E (P)	Antipolo	02538KBFR9	Fruit	25.7	Diarrhea; cooked (Tan. (1980); Stuart. (2017))
*Artocarpus camansi* Blanco (Moraceae)	10.6819242N, 124.8001064E (P)	Kamansi	02512KBFR1	Bark	13.3	Diarrhea; cooked (Tan. (1980); Stuart. (2017))
*Artocarpus elasticus* Reinw. ex Blume (Moraceae)	10.7435939N, 124.8019275E (P)	Terap	02539KBFRA	Bark	11.8	Dysentery (Lim, 2012; interviewed herbalist)
*Artocarpus odoratissimus* Blanco (Moraceae)	10.7436072N, 124.8017989E (P)	Marang	02540KBFR2	Fruit	22.9	Diarrhea (Lim. (2012); interviewed herbalist)
*Bauhinia malabarica* Roxb. (Leguminosae)	12.4428908N, 107.1592217E (C)	Choeung Koo	02562KBFR6	Bark and leaves	14.7 and 11.2	Diarrhea and abdominal pain; alcohol maceration or decoction (Chassagne et al. (2016))
*Breynia cernua* (Poir.) Müll.Arg. (Phyllantaceae)	9.8153556N, 124.3597258E (P)	Mutang-Ulang	02541KBFR3	Bark	10.6	Dysentery; infusion (van Valkenburg and Bunyapraphatsara (2001))
*Breynia vitis-idaea* (Burm.f.) C.E.C.Fisch. (Phyllanthaceae)	11.5627122N, 104.9167906E (C)	Phnek Preab	02563KBFR7	Wood with bark	9.9	Dysentery; infusion (Kham, 2004)
*Commelina communis* L. (Commelinaceae)	10.6159294N, 124.9272431E (P)	Alibangon	02542KBFR4	Whole plant	13.3	Diarrhea (van Valkenburg and Bunyapraphatsara (2001))
*Cyathula prostrata* (L.) Blume (Amaranthaceae)	10.7433806N, 124.8001225E (P)	Dayang	02543KBFR5	Whole plant	12.8	Dysentery and cholera; decoction or infusion (van Valkenburg and Bunyapraphatsara (2001); Stuart. (2017))
*Diplazium esculentum* (Retz.) Sw. (Athyriaceae)	10.7577433N, 124.7975153E (P)	Paco	02545KBFR7	Rhizome	5.4	Diarrhea and dysentery; pulverization and cold water maceration (Stuart. (2017); interviewed herbalist)
*Ehretia microphylla* Lam. (Boraginaceae)	10.7442369N, 124.7897825E (P)	Tsaang-Gubat	02489KBFRE	Leaves	15.3	Diarrhea, dysentery, and abdominal pain; decoction or infusion (8 tbsp of chopped leaves in 2 glasses) (de Padua et al. (1999); Stuart. (2017))
*Emilia sonchifolia* (L.) DC. ex DC. (Compositae)	10.7407072N, 124.8002914E (P)	Tagulinaw	02520KBFR0	Whole plant	20.9	Diarrhea, dysentery, and enteritis; decoction (6–15 gDW) (Tan. (1980); Stuart. (2017))
*Helicteres angustifolia* L. (Malvaceae)	12.3963028N, 107.1938622E (C)	Sambok Cheas	02564KBFR8	Root	9.2	Diarrhea, dysentery, and abdominal pain; decoction (Chassagne et al. (2016))
*Hyptis capitata* Jacq. (Lamiaceae)	10.7590292N, 124.8020589E (P)	Botonesan	02546KBFR8	Whole plant	10.1	Gastrointestinal problems; decoction (Lemmens and Bunyapraphatsara (2003))
*Ixora nigricans* R.Br. ex Wight and Arn. (Rubiaceae)	13.7291931N, 107.0113667E (C)	Phka Mochul Pich	02565KBFR9	Leaves	10.8	Dysentery and abdominal pain (Kham. (2004))
*Kyllinga brevifolia* Rottb. (Cyperaceae)	11.0610592N, 124.7009597E (P)	Pugo-Pugo	02544KBFR6	Whole plant	11.4	Diarrhea (de Padua et al. (1999); Stuart. (2017))
*Lagerstroemia cochinchinensis* Pierre ex Gagnep. (Lythraceae)	13.4692872N, 105.8909203E (C)	Sralao	02566KBFRA	Bark	2.8	Diarrhea; decoction (Chassagne et al. (2016))
*Leea indica* (Burm. f.) Merr. (Vitaceae)	11.5627122N, 104.9167906E (C)	Kdaing Baay	02567KBFRB	Root	8.3	Diarrhea, dysentery, digestive and intestinal complaints; decoction or infusion (Kham. (2004))
*Melastoma dodecandrum* Lour. (Melastomataceae)	12.4089644N, 107.3133011E (C)	Unknown	02568KBFRC	Bark and leaves with flower buds	12.7 and 9.9	Diarrhea (van Duong. (1993))
*Melastoma saigonense* (Kuntze) Merr. (Melastomataceae)	11.5627122N, 104.9167906E (C)	Baay Nhenh	02569KBFRD	Wooden stem and leaves with flower buds	7.3 and 17.3	Diarrhea (Chassagne et al. (2016))
*Parkia javanica* (Lam.) Merr. (Leguminosae)	10.7448892N, 124.8059375E (P)	Kupang	02547KBFR9	Bark	25.7	Diarrhea and dysentery; decoction (Tan. (1980); Stuart. (2017))
*Pentacme siamensis* (Miq.) Kurz (Dipterocarpaceae)	13.4474300N, 105.8756317E (C)	Raing Phnom	02571KBFR6	Bark	5.8	Diarrhea (Chassagne et al. (2016)
*Picrasma javanica* Blume (Simaroubaceae)	10.7438825N, 124.8039956E (P)	Manunggal	02548KBFRA	Bark	6.3	Digestive and abdominal pain; decoction (Langenberger et al. (2008))
*Pseudelephantopus spicatus* (Juss. ex Aubl.) Rohr (Compositae)	9.8110686N, 124.3551231E (P)	Kokunbanog	02553KBFR6	Whole plant	12.5	Diarrhea; decoction (Langenberger et al. (2008))
*Rourea minor* (Gaertn.) Alston (Connaraceae)	12.3965372N, 107.1933392E (C)	Unknown	02570KBFR5	Leaves	11.4	Diarrhea (Chassagne et al. (2016))
*Tabernaemontana pandacaqui* Lam. (Apocynaceae)	14.1667808N, 121.2143336E (P)	Pandakaking-Puti	02503KBFR1	Bark	10.1	Gastroenteritis (Tan. (1980))
*Triumfetta bartramia* L. (Malvaceae)	10.7467864N, 124.8152500E (P)	Kulutkulutan	02554KBFR7	Root	14.9	Diarrhea and intestinal ulcers (van Valkenburg and Bunyapraphatsara (2001); Stuart. (2017))

C, Cambodia; P, Philippines.

### Preparation of Plant Extracts

Although the most common procedures of processing antidiarrheal plants in Cambodia and the Philippines are decoction and infusion (Table 1), ethanol was selected for the extraction of plant samples since it is an efficient solvent for herbal drugs with a well-established tradition in herbal medicine (Kelber et al., 2016). With the aim of preventing possible loss or changes of active constituents due to storage of plant samples, the extraction was performed immediately after their arrival in the Czechia. Each dried sample was homogenized into powder using Grindomix mill (Retsch, Haan, Germany), and 15 g of dry matter was extracted in 450 ml 80% ethanol (Penta, Prague, Czechia) for 24 h at room temperature using a laboratory shaker (GFL3005, GFL, Burgwedel, Germany). Therefore, the drug extract ratio was 1:30. Extracts were subsequently filtered and concentrated using a rotary vacuum evaporator (R-200, Buchi Labortechnik, Flawil, Switzerland) *in vacuo* at 40°C. According to the recommendations of Cos et al. (2006), the dried residue was finally diluted in 100% dimethylsulfoxide (DMSO) (Penta, Prague, Czechia) to obtain stock solutions with a final concentration of 51.2 mg/ml and stored at −20°C until their use. Some of the extracts were not completely soluble in other solvents, such as distilled water. Yields (%) of the dried residues are shown in Table 1.

### Bacterial Strains and Media

The intestinal bacterial type strains were obtained from the American Type Culture Collection (ATCC, Rockville, MD, United States), Czech Collection of Microorganisms (CCM, Brno, Czechia), German Collection of Microorganisms and Cell Cultures (DSMZ, Braunschweig, Germany), and National Collection of Type Cultures (NCTC, London, United Kingdom).

In accordance with the diversity of diarrheagenic gram-positive and gram-negative bacteria responsible for globally distributed foodborne, waterborne, and nosocomial infections (Diniz-Santos et al., 2006; Rajkovic et al., 2020), the following 12 strains were used in this study: *B. cereus* (ATCC 14579), *C. difficile* (DSMZ 12056), *C. perfringens* (DSMZ 11778), *E. faecalis* (ATCC 29212), *E. coli* (ATCC 25922), *E. coli* 0175:H7 (NCTC 12900), *L. monocytogenes* (ATCC 7644), *Shigella flexneri* (ATCC 12022), *Salmonella enterica* ssp. *enterica* serovar Enteritidis (ATCC 13076), *S. enterica* ssp. *enterica* serovar Typhimurium (ATCC 14028), *V. parahaemolyticus* (ATCC 17802), and *Y. enterocolitica* (ATCC 9610). The above-mentioned strains were considered as obligate or facultative pathogens. The following six bacterial strains, which belong to the dominant bacterial phyla in the human gut and exhibit probiotic functions (Behnsen et al., 2013), were used in this study: *Bifidobacterium adolescentis* (DSMZ 20087), *Bifidobacterium animalis* spp. *lactis* (DSMZ 10140), *Bifidobacterium breve* (ATCC 15700), *Lactobacillus casei* (DSMZ 20011), *Lactobacillus reuteri* (CCM 3625), and *Lactobacillus rhamnosus* (CCM 7091). All these strains were considered beneficial gut bacteria.

As the maintenance and growth medium, Mueller-Hinton Broth (Oxoid, Basingstoke, United Kingdom) was used for the majority of bacteria that grow aerobically (*E. faecalis* supp. 1% glucose, *V. parahaemolyticus* supp. 3% NaCl). *Y. enterocolitica* was stored and cultured in Brain Heart Infusion Broth (Oxoid, Basingstoke, United Kingdom). Bifidobacteria and lactobacilli were maintained and cultured in Wilkins-Chalgren Broth (Oxoid, Basingstoke, United Kingdom) supplemented with 5 g/L soya peptone and 0.5 g/L cysteine. Although the same growth medium was used for clostridia, they were stored in cooked meat medium (both from Oxoid, Basingstoke, United Kingdom) at room temperature. The standard safety guidelines for handling microorganisms were followed. Therefore, all items, such as culture tubes, syringes, and gloves, were discarded in the biohazard autoclave bag after every use.

### Cell Cultures

One representative of normal intestinal cell line (FHs 74 Int [ATCC CCL 241]) and two of cancer intestinal cell lines (Caco-2 [ATCC HTB 37]) and HT-29 [ATCC HTB 38]) were purchased from ATCC (Rockville, MD, United States). Normal cells were cultured in Hybri-Care medium supplemented with 10% fetal bovine serum, 1% sodium bicarbonate, 1% nonessential amino acids, 30 ng/ml of epidermal growth factor, and 1% penicillin-streptomycin solution (10,000 units/ml and 100 mg/ml, respectively). The cancer cells were cultured in Eagle’s Minimum Essential Medium (EMEM) supplemented with 1% sodium pyruvate, 10% fetal bovine serum, 1% sodium bicarbonate, 1% nonessential amino acids, and 1% penicillin-streptomycin solution (10,000 units/ml and 100 mg/ml, respectively) (all purchased from Biowest, Nuaille, France). The cultures were incubated at 37°C and 5% CO_2_. The culture medium was replaced every 2–3 days, and cells were passaged every 7 days.

### Antibacterial Assay

Initially, all 35 extracts (Table 1) were evaluated for their antibacterial activities against the pathogenic strains. Those showing any inhibitory action were subsequently tested against the probiotic strains. The growth-inhibitory activities against aerobic and anaerobic bacteria were evaluated using the broth microdilution method using 96-well microtiter plates, following the protocols of (Clinical and Laboratory Standards Institute, 2021) and Hecht (1999), respectively. For the effective assessment of the anti-infective potential of natural products, slight modifications were implemented as described by Cos et al. (2006).

Prior to testing, the strains that grow aerobically were subcultured in the appropriate media at 37°C (*Y. enterocolitica* at 30°C) for 24 h. Bifidobacteria, clostridia, and lactobacilli were cultured at 37°C for 48 h using Whitley A35 Anaerobic Workstation (Don Whitley Scientific, Bingley, United Kingdom). The anaerobic conditions were created by supplying a standard anaerobic gas mixture of 10% H_2_, 10% CO_2_, and 80% N_2_ (Linde Gas, Prague, Czechia).

The extracts were diluted twofold in appropriate growth media (initial concentration of 512 μg/ml) using the Freedom EVO 100 automated pipetting platform (Tecan, Männedorf, Switzerland) and multichannel pipette (Eppendorf, Hamburg, Germany) in case of aerobic and anaerobic bacteria, respectively. After the optimalization of bacterial cultures to inoculum density of 1.5 × 10^8^ CFU/ml by 0.5 McFarland standard using Densi-La-Meter II (Lachema, Brno, Czechia), the cultures were inoculated in 96-well plates (5 μl/well). Bacterial cultures in microplates were incubated by employing the same protocols as used for their cultivation prior to the test. The optical density of the cultures was measured at 405 nm (OD_450 nm_) using a Cytation 3 Imaging Reader (BioTek, Winooski, VT, United States) before and after the growth period.

The lowest concentration (μg/ml) of the extracts that inhibited the bacterial growth by ≥80% was defined as the minimum inhibitory concentration (MIC). Ciprofloxacin (Sigma-Aldrich, Prague, Czechia), an antibiotic commonly recommended for the treatment of infectious diarrhea (Casburn-Jones, 2004), was dissolved in distilled water and used as a positive control drug. All tests were performed as three independent experiments, each conducted in triplicate. The mode and median were used for the final MIC value calculation when the triplicate endpoints were within the two- and three-dilution ranges, respectively. The antibacterial activities were classified as strong (MICs ≤64 μg/ml), moderate (MICs = 128–256 μg/ml), and weak (MIC = 512 μg/ml) (Kokoska et al., 2019). As a result of experiments performed without dissolved extracts and ciprofloxacin (Sigma-Aldrich, Prague, Czechia), their respective solvents, namely, DMSO (Sigma-Aldrich, Prague, Czechia) and distilled water, did not inhibit bacterial growth of any strain at the tested concentrations (≤1%).

### Cytotoxicity Assay

The antiproliferative activities of the extracts that showed some inhibitory action against the tested bacteria were further assessed using the modified thiazolyl blue tetrazolium bromide (MTT) cytotoxicity assay developed by Mosmann (1983). Cancer (2.5 × 10^3^) and normal intestinal (2.5 × 10^5^) cells were seeded in a 96-well microtiter plate for 24 h. Cells were incubated with twofold serially diluted plant extracts (0.25–512 μg/ml) for 72 h. Next, the cells were incubated with MTT reagent (1 mg/ml) (Sigma-Aldrich, Prague, Czechia) in EMEM or Hybri-Care medium for an additional 2 h at 37°C and 5% CO_2_. The medium with MTT was removed, and the intracellular formazan product was dissolved in 100 μl DMSO. The absorbance was measured at 555 nm using a Tecan Infinite M200 spectrometer (Tecan, Männedorf, Switzerland), and the percentage of viability was calculated when compared to an untreated control.

The antiproliferative activity of the tested plant extracts was represented as half-maximal inhibitory concentration (IC_50_; μg/ml). The colon cancer chemotherapeutic drug 5-fluorouracil (Sigma-Aldrich, Prague, Czechia) was used as a positive control (Fuente et al., 2020). Three independent experiments (two replicates each) were performed for every test. Data are presented as mean ± standard deviation. The antiproliferative activity was evaluated as follows: cytotoxic (IC_50_ values ≤100 μg/ml), moderately cytotoxic (IC_50_ values = 100–400 μg/ml), and weakly cytotoxic (IC_50_ values = 401–512 μg/ml) (Srisawat et al., 2013). The solvents did not affect the viability of normal and cancer intestinal cell lines at the tested concentration (≤1%).

### Calculations

For comparison of microbiological and toxicological data, 80% bacterial growth inhibition (IC_80_) was calculated as equivalent to the MIC endpoint (Houdkova et al., 2018). Subsequently, *x̄*-MIC, *x̄*-IC_50_, and *x̄*-IC_80_ values (±standard deviations) were calculated to quantify the inhibitory activity of the tested plant extracts against pathogenic/beneficial bacteria and intestinal cancer/normal cells. Subsequently, the selectivity index (SI) was calculated between normal intestinal cells and pathogenic strains (SIa), beneficial and pathogenic strains (SIb), normal and cancer intestinal cells (SIc), and beneficial strains and cancer intestinal cells (SId) using the following formulas where X1 = IC_80_ against normal intestinal cells; X2 = *x̄*-MIC against beneficial strains; X3 = IC_50_ against normal intestinal cells; Y1 = *x̄*-MIC against pathogenic strains; Y2 = *x̄*-IC_50_ against cancer intestinal cells; and Y3 = IC_80_ against cancer intestinal cells:

SIa = log (X1/Y1), (1)

SIb = log (X2/Y1), (2)

SIc = log (X3/Y2), (3)

SId = log (X2/Y3). (4)

The SI values >0 and <0 indicate selective toxicity against pathogenic strains/cancer cell lines and beneficial strains/normal cell lines, respectively.

## Results

### Antibacterial Activity

#### Diarrheagenic Bacterial Pathogens

Considering the antibacterial activity against the pathogens, 16 of 35 tested extracts revealed a growth-inhibitory effect on at least one of these bacterial strains. While *B. cereus*, *C. difficile*, *E. coli,* and *V. parahaemolyticus* were the most susceptible bacteria inhibited by the highest number of extracts, none of the extracts exerted activity against *E. coli* O157:H7 and *S. flexneri*. The MICs (32–512 μg/ml) of all 16 plant extracts for the diarrheagenic bacterial pathogens are presented in Table 2.

**TABLE 2:** *In vitro* selective inhibitory activities of ethanolic extracts of Cambodian and Philippine plants against intestinal bacteria and cells.

Cultures tested			Plant species with their parts and positive antibiotic and anticancer control																	
			*AP(w)*	*AG(l)*	*AT(l)*	*AB (f)*	*AC(b)*	*BM(b)*	*BV(wb)*	*DE(r)*	*EM(l)*	*IN(l)*	*LC(b)*	*MD(b)*	*MD(lf)*	*MS(lf)*	*PJ(b)*	*PS(b)*	CIP	5-FU
Bacterial strain/MIC (µg/ml)		BC	-^a^	512	64	64	256	512	-	-	512	-	-	-	-	512	-	256	1	nd
		CD	512	512	512	32	128	-	-	-	512	512	-	-	512	512	-	-	16	nd
		CP	-	512	512	32	256	-	-	-	-	-	-	-	-	-	-	-	1	nd
		EF	-	-	-	128	-	-	-	-	-	-	-	-	-	-	-	-	2	nd
		EC	512	-	-	-	-	256	256	512	-	-	-	256	-	-	256	256	0.062	nd
		ECS	-	-	-	-	-	-	-	-	-	-	-	-	-	-	-	-	0.016	nd
		LM	-	-	128	256	-	-	-	-	-	-	-	-	-	512	-	512	4	nd
		SF	-	-	-	-	-	-	-	-	-	-	-	-	-	-	-	-	0.016	nd
		SE	-	-	-	-	-	-	-	-	-	-	-	-	-	-	-	256	0.031	nd
		ST	-	-	-	-	-	-	256	-	-	-	-	-	512	-	-	-	0.031	nd
		VP	-	256	-	-	-	512	-	-	512	-	512	512	512	512	-	-	0.062	nd
		YE	-	-	-	-	-	-	-	-	-	-	-	-	-	-	-	-	0.125	nd
		*x̄-*PB ± SD	938.7 ± 191	832 ± 279	784 ± 390	640 ± 458	821.3 ± 352	874.7 ± 266	896 ± 286	981.3 ± 142	896 ± 222	981.3 ± 142	981.3 142±	917.3 ± 244	896 ± 222	853.3 ± 241	960 ± 212	789.3 ± 338	2 ± 6	nd
		BA	64	512	256	16	-	-	-	-	512	512	-	-	256	512	-	-	8	nd
		BB	-	64	64	16	512	-	-	-	128	128	-	-	-	256	128	-	64	nd
		BLC	-	**-**	256	16	-	-	-	-	256	512	-	-	-	512	-	-	32	nd
		LC	512	256	128	16	256	-	-	-	128	128	-	-	-	512	512	512	32	nd
		LR	-	-	256	16	**-**	-	-	-	-	-	-	-	-	-	-	-	32	nd
		LRM	-	-	128	16	512	-	-	-	512	-	-	-	-	-	-	-	4	nd
		*x̄-*BB ± SD	778.7 ± 370	650.7 ± 395	181.3 ± 78	16 ± 0	725.3 ± 311	1,024 ± 0	1,024 ± 0	1,024 ± 0	426.7 ± 311	554.7 ± 367	1,024 ± 0	1,024 ± 0	896 ± 286	640 ± 286	789.3 ± 350	938.7 ± 191	29 ± 20	nd
Cell line (µg/ml)	IC_50_ ± SD	HT-29	130.52 ± 2.57	96.53 ± 12.41	82.19 ± 17.22	53.70 ± 16.02	84.77 ± 4.20	35.195 ± 5.32	81.79 ± 20.79	-	130.89 ± 13.99	125.55 ± 13.92	37.89 ± 2.68	248.56 ± 23.13	210.85 ± 16.83	49.75 ± 3.53	155.87 ± 41.79	51.98 ± 19.79	88.81 ± 13.44	6.35 ± 2.07
		Caco-2	148.96 ± 17.14	78.42 ± 23.18	33.82 ± 10.57	79.41 ± 6.90	48.40 ± 0.93	-	-	-	52.49 ± 8.81	135.60 ± 3.11	122.86 ± 13.16	193.73 ± 1.61	77.57 ± 10.22	87.40 ± 19.18	121.26 ± 15.34	-	90.87 ± 17.98	181.79 ± 151.51
		*x̄-*CC ± SD	139.7 ± 9.2	87.5 ± 9	58 ± 24.2	66.5 ± 12.8	66.5 ± 18	529.6 ± 494	552.9 ± 471	-	91.7 ± 39.2	130.6 ± 5	80.4 ± 42.5	221.1 ± 27.4	144.2 ± 66.6	68.575 ± 18.8	138.6 ± 17.3	538 ± 486	89.84 ± 1	94 ± 88
		FHs 74 Int	297.39 ± 22.54	118.76 ± 36.04	45.50 ± 7.29	273.32 ± 7.50	68.23 ± 12.39	158.36 ± 23.75	68.75 ± 8.89	-	303.41 ± 18.00	243.50 ± 21.92	282.05 ± 0.57	-	368.07 ± 30.00	195.19 ± 8.94	342.62 ± 3.54	-	58.90 ± 4.27	492.43 ± 22.92
	IC_80_ ± SD	HT-29	130.50 ± 12.06	-	498.17 ± 16.74	-	356.20 ± 19.04	-	-	-	-	-	-	351.26 ± 43.24	378.50 ± 34.93	143.70 ± 16.74	-	-	181.53 ± 15.05	367.26 ± 0.57
		Caco-2	-	-	-	-	-	-	-	-	-	-	-	-	-	-	-	-	-	-
		*x̄-*CC ± SD	577.25 ± 446.75	-	761.1 ± 263	-	690.1 ± 333.9	-	-	-	-	-	-	687.63 ± 336.37	701.25 ± 322.75	583.85 ± 440.15	-	-	602.77 ± 421.23	695.63 ± 328.37
		FHs 74 Int	-	-	-	-	264.64 ± 6.82	-	-	-	-	-	-	-	-	-	-	-	83.37 ± 8.60	-
SI		(a)	0.04	0.09	0.12	0.2	−0.49	0.07	0.06	0.02	0.06	0.02	0.02	0.05	0.06	0.08	0.03	0.11	1.62	nd
		(b)	−0.1	−0.1	−0.6	−1.6	−0.1	0.1	0.1	0.02	−0.3	−0.2	0.02	0.05	0	−0.1	−0.04	0.1	1.2	nd
		(c)	0.3	0.1	−0.1	0.6	0.01	−0.5	−0.9	0	0.5	0.3	0.5	0.7	0.4	0.5	0.4	0.3	−0.2	0.4
		(d)	0.13	−0.2	−0.62	−1.81	0.02	0	0	0	−0.38	−0.27	0	0.17	0.11	0.04	−0.11	−0.04	−1.32	nd

MIC, minimum inhibitory concentration; IC_50_, half-maximal inhibitory concentration; IC_80_, 80% inhibitory concentration of proliferation; SD, standard deviation; ^a^Not active (MIC/IC_50/80_ >512 μg/ml, the value 1,024 μg/ml was used for average calculation); nd: no data. AP(w), Aganonerion polymorphum Spire (whole plant); AG(l), Acalypha grandis Benth. (leaves); AT(l), Ancistrocladus tectorius (Lour.) Merr. (leaves); AB (f), Artocarpus blancoi (Elmer) Merr. (fruit); AC(b), Artocarpus camansi Blanco (bark); BM(b), Bauhinia malabarica Roxb. (bark); BV(wb), Breynia vitis-idaea (Burm.f.) C.E.C.Fisch. (wood with bark); DE(r), Diplazium esculentum (Retz.) S roots); EM(l), Ehretia microphylla Lam. (leaves); IN(l), Ixora nigricans R.Br. ex Wight and Arn. (leaves); LC(b), Lagerstroemia cochinchinensis Pierre ex Gagnep. (bark); MD(b), Melastoma dodecandrum Lour. (bark); MD(lf), Melastoma dodecandrum Lour. (leaves with flower buds); MS(lf), Melastoma saigonense (Kuntze) Merr. (leaves with flower buds); PJ(b), Picrasma javanica Blume (bark); PS(b), Pentacme siamensis (Miq.) Kurz; CIP, ciprofloxacin; 5-FU, 5-fluorouracil. BC, Bacillus cereus; CD, Clostridium difficile; CP, Clostridium perfringens; EF, Enterococcus faecalis; EC, Escherichia coli; ECS, E. coli 0,175:H7; LM, Listeria monocytogenes; SF, Shigella flexneri; SE, Salmonella Enteritidis; ST, Salmonella Typhimurium; VP, Vibrio parahaemolyticus; YE, Yersinia enterocolitica; BA, Bifidobacterium adolescentis; BB, Bifidobacterium breve; BLC, Bifidobacterium animalis spp. lactis, LC, Lactobacillus casei; LR, Lactobacillus reuteri; LRM, Lactobacillus rhamnosus; x̄-PB, mean MIC for pathogenic bacteria; x̄-BB, mean MIC for beneficial bacteria; x̄-CC, mean IC_50/80_ for intestinal cancer cells; FHs 74 Int (intestinal normal cells); SI (selectivity index): (a) normal cells/diarrheagenic bacteria, (b) beneficial bacteria/diarrheagenic bacteria, (c) normal cells/cancer cells, and (d) beneficial bacteria/cancer cells.

There were four extracts showing promising antibacterial actions against multiple pathogenic bacteria, especially the gram-positive strains. Namely, the fruit extract of *Artocarpus blancoi* (Elmer) Merr. (Moraceae) inhibited *B. cereus* and both clostridia at MICs 64 and 32 μg/ml, respectively. This plant was also moderately active against *E. faecalis* (MIC = 128 μg/ml) and *L. monocytogenes* (MIC = 256 μg/ml). Similarly, the leaf extract of *Ancistrocladus tectorius* (Lour.) Merr. (Ancistrocladaceae) revealed a strong inhibitory effect on *B. cereus* (MIC = 64 μg/ml) and moderate activity against *L. monocytogenes* (MIC = 128 μg/ml). However, it produced only weak inhibitory action against both clostridia (MICs = 512 μg/ml). Next, bark extract of *Artocarpus camansi* Blanco (Moraceae) inhibited *B. cereus* and both clostridia at MICs ranging from 128 to 256 μg/ml. Although the antibacterial activities of bark extract of *Pentacme siamensis* (Miq.) Kurz (Dipterocarpaceae) were rather moderate, it exerted inhibitory action against several gram-positive as well as gram-negative pathogenic strains. Namely, it inhibited *B. cereus*, *E. coli,* and *S.* Enteritidis at MICs of 256 μg/ml and *L. monocytogenes* at MIC of 512 μg/ml.

Additionally, there were five more plant extracts exerting moderate activity (MIC = 256 μg/ml) against a single gram-negative strain (Table 2). Namely, *Bauhinia malabarica* Roxb. (Leguminosae) (bark), *Breynia vitis-idaea* (Burm.f.) C.E.C.Fisch. (Phyllanthaceae), *Melastoma dodecandrum* Lour. (Melastomataceae) (bark), and *Picrasma javanica* Blume (Simaroubaceae) inhibited *E. coli*; *B. vitis-idaea* (Burm.f.) C.E.C.Fisch. inhibited *S*. Typhimurium; and *Acalypha grandis* Benth. (Euphorbiaceae) inhibited *V. parahaemolyticus*. Finally, *Aganonerion polymorphum* Spire (Apocynaceae), *Diplazium esculentum* (Retz.) Sw. (Athyriaceae), *Ehretia microphylla* Lam. (Boraginaceae), *Ixora nigricans* R.Br. ex Wight and Arn. (Rubiaceae), *Lagerstroemia cochinchinensis* Pierre ex Gagnep. (Lythraceae), *Melastoma dodecandrum* Lour. (Melastomataceae) (leaves with flower buds), and *Melastoma saigonense* (Kuntze) Merr. (Melastomataceae) (leaves with flower buds) produced only weak inhibitory actions at MICs of 512 μg/ml (Table 2).

The remaining 19 extracts of *Acanthus ebracteatus* Vahl (Acanthaceae)*, Aporosa villosa* (Lindl.) Baill. (Phyllanthaceae)*, Artocarpus elasticus* Reinw. ex Blume (Moraceae)*, Artocarpus odoratissimus* Blanco (Moraceae)*, B. malabarica* Roxb. (leaves), *Breynia cernua* (Poir.) Müll.Arg. (Phyllantaceae)*, Commelina communis* L. (Commelinaceae)*, Cyathula prostrata* (L.) Blume (Amaranthaceae)*, Emilia sonchifolia* (L.) DC. ex DC. (Compositae)*, Helicteres angustifolia* L. (Malvaceae)*, Hyptis capitata* Jacq. (Lamiaceae)*, Kyllinga brevifolia* Rottb. (Cyperaceae)*, Leea indica* (Burm. f.) Merr. (Vitaceae)*, M. saigonense* (Kuntze) Merr. (wooden stem and leaves), *Parkia javanica* (Lam.) Merr. (Leguminosae)*, Pseudelephantopus spicatus* (Juss. ex Aubl.) Rohr (Compositae)*, Rourea minor* (Gaertn.) Alston (Connaraceae)*, Tabernaemontana pandacaqui* Lam. (Apocynaceae)*,* and *Triumfetta bartramia* L. (Malvaceae) did not show any inhibitory action; thus, they have not been further discussed.

#### Beneficial Gut Bacteria

Subsequently, 16 extracts that exerted growth-inhibitory effect against diarrheagenic pathogens were verified for their safety to beneficial bacteria. The final MICs are presented in Table 2. Five extracts, namely, *B. malabarica* Roxb., *B. vitis-idaea* (Burm.f.) C.E.C.Fisch, *D. esculentum* (Retz.) Sw., *L. cochinchinensis* Pierre ex Gagnep., and *M. dodecandrum* Lour. (bark), did not have any inhibition of these strains (MICs >512 μg/ml), suggesting their harmless effect on gut commensals.

The remaining 11 extracts affected to some degree the growth of beneficial gut bacteria, particularly of bifidobacteria and *L. casei* (Table 2). The single strain was inhibited by *P. siamensis* (Miq.) Kurz (*L. casei*) and leaf with flower bud of *M. dodecandrum* Lour. (*B. adolescentis*) at MICs of 256 and 512 μg/ml, respectively. Moreover, *P. javanica* Blume inhibited *B. breve* (MIC = 128 μg/ml) and *L. casei* (MIC = 512 μg/ml). Although *A. polymorphum* Spire significantly affected the growth of *B. adolescentis* (MIC = 64 μg/ml), the remaining probiotic strains were rather resistant toward this extract (MICs ≥512 μg/ml). Three and four probiotic bacteria were inhibited (MICs = 256–512 μg/ml) by *A. camansi* Blanco and *M. saigonense* (Kuntze) Merr., respectively. At MICs ranging from 128 to 512 μg/ml (Table 2), *E. microphylla* Lam. and *I. nigricans* R.Br. ex Wight and Arn. affected the growth of the majority of beneficial strains. Although half of the bacteria were not inhibited by *A. grandis* Benth., this extract inhibited *B. breve* at low MIC (64 μg/ml). Finally, all six strains were inhibited by *A. blancoi* (Elmer) Merr. and *A. tectorius* (Lour.) Merr. Whereas the former uniformly affected the growth at very low MICs (16 μg/ml), the latter inhibited *B. breve* (MIC = 64 μg/ml) only.

### Cytotoxic Effect

The outcomes of the MTT assay for all 16 antibacterially active plant extracts against normal and cancer intestinal cells are presented in Table 2. With the exception of *D. esculentum* (Retz.) Sw. (IC_50_ values > 512 μg/ml), all the 16 extracts produced a certain antiproliferative effect on at least one of the tested cell lines (IC_50_ values = 33.82 ± 10.57–368.07 ± 30.00 μg/ml).

#### Normal Intestinal Cells

Considering the toxicity to normal intestinal cells (FHs 74 Int), *M. dodecandrum* Lour. (bark) and *P. siamensis* (Miq.) Kurz did not show inhibitory action at the concentrations tested (IC_50_ > 512 μg/ml) (Table 2). Moderate toxicity was shown by *A. polymorphum* Spire*, A. grandis* Benth.*, A. blancoi* (Elmer) Merr.*, B. malabarica* Roxb.*, E. microphylla* Lam.*, I. nigricans* R.Br. ex Wight and Arn.*, L. cochinchinensis* Pierre ex Gagnep.*, M. dodecandrum* Lour. (leaves with flower buds), *M. saigonense* (Kuntze) Merr., and *P. javanica* Blume at IC_50_ values ranging from 118.76 ± 36.04 to 368.07 ± 30.00 μg/ml. Finally, the extracts of *A. tectorius* (Lour.) Merr., *A. camansi* Blanco, and *B. vitis-idaea* (Burm.f.) C.E.C.Fisch. were shown to be cytotoxic (IC_50_ values = 45.50 ± 7.29, 68.23 ± 12.39 and 68.75 ± 8.89 μg/ml, respectively).

#### Cancer Intestinal Cells

Regarding the antiproliferative activities against cancer intestinal cells (Table 2), the plants producing strong effects on Caco-2 (IC_50_ values = 33.82 ± 10.57–87.40 ± 19.18 μg/ml) have been ordered as follows: *A. tectorius* (Lour.) Merr., *A. camansi* Blanco, *E. microphylla* Lam., *M. dodecandrum* Lour. (leaves with flower buds), *A. grandis* Benth., *A. blancoi* (Elmer) Merr., and *M. saigonense* (Kuntze) Merr. With the exception of moderately cytotoxic *E. microphylla* Lam. and *M. dodecandrum* Lour. (leaves with flower buds), the same plant extracts with the addition of *B. malabarica* Roxb., *B. vitis-idaea* (Burm.f.) C.E.C.Fisch.*, L. cochinchinensis* Pierre ex Gagnep., and *P. siamensis* (Miq.) Kurz also produced strong antiproliferative effect on HT-29 (IC_50_ values = 35.195 ± 5.32–96.53 ± 12.41 μg/ml) (Table 2). A moderate cytotoxic effect on both these cancer cell lines was then shown by *A. polymorphum* Spire, *I. nigricans* R.Br. ex Wight and Arn., *M. dodecandrum* Lour. (bark), and *P. javanica* Blume (IC_50_ values = 121.26 ± 15.34–248.56 ± 23.13 μg/ml). The majority of extracts revealed higher activities against Caco-2 than that of 5-fluorouracil (IC_50_ = 181.79 ± 151.51 μg/ml).

### Selective Toxicity

The calculated mean values for pathogenic/beneficial bacteria, cancer cells (*x̄*-MIC, *x̄*-IC_50_, and *x̄*-IC_80_), and derived SIs are presented in Table 2. Comparing the concentrations inhibiting 80% of growth for pathogenic bacteria and normal intestinal cells, the antibacterially active extracts were shown to be relatively safe (SIa values = 0.02–0.2; IC_80_ values >512 μg/ml) except *A. camansi* Blanco (SIa = −0.49; IC_80_ = 264.64 ± 6.82 μg/ml). Selective antibacterial effect (SIb values = 0.1) with relative safety for beneficial strains was shown by *B. malabarica* Roxb., *B. vitis-idaea* (Burm.f.) C.E.C.Fisch., and *P. siamensis* (Miq.) Kurz (Table 2). However, none of the selective effects were as significant as in the case of ciprofloxacin (SIb = 1.2). Other extracts did not show any noticeable selectivity or were comparably more harmful to beneficial bacteria, especially *A. blancoi* (Elmer) Merr., and *A. tectorius* (Lour.) Merr. (SIb values = −1.6 and −0.6, respectively). Regarding the selective antiproliferative effects against cancer intestinal cells, *A. blancoi* (Elmer) Merr.*, E. microphylla* Lam.*, L. cochinchinensis* Pierre ex Gagnep.*, M. dodecandrum* Lour. (bark), and *M. saigonense* (Kuntze) Merr. revealed higher selectivity (SIc values = 0.5–0.7) than that of 5-fluorouracil (SIc = 0.4) (Table 2). Other extracts produced either the same or lower degree of selective effects than that of this cytotoxic drug, whereas *A. tectorius* (Lour.) Merr., *B. malabarica* Roxb., and *B. vitis-idaea* (Burm.f.) C.E.C.Fisch. were relatively more toxic to normal intestinal cells (SIc values = −0.9 to −0.1). The probiotic strains were not affected by the antiproliferative concentrations of *A. polymorphum* Spire (SId = 0.13), mainly because of moderate inhibition of HT-29 (IC_80_ = 130.50 ± 12.06 μg/ml) (Table 2). Interestingly, the extract of *P. siamensis* (Miq.) Kurz produced noticeable selective actions combining antibacterial and antiproliferative effects on pathogenic bacteria and intestinal cancer cells without affecting beneficial bacteria and normal intestinal cells.

## Discussion

In the present study, 16 of 35 tested extracts revealed *in vitro* growth-inhibitory effect on the diarrheagenic bacterial pathogens, especially *A. blancoi* (Elmer) Merr., *A. camansi* Blanco, *A. tectorius* (Lour.) Merr., and *P. siamensis* (Miq.). Except *A. camansi* Blanco, the antibacterially active concentrations of the three were nontoxic to normal intestinal cells. Among the 16 extracts, *A. blancoi* (Elmer) Merr., *E. microphylla* Lam., *L. cochinchinensis* Pierre ex Gagnep., *M. saigonense* (Kuntze) Merr., and *P. siamensis* (Miq.) Kurz also revealed a strong selective antiproliferative effect against intestinal cancer lines. The extract of *P. siamensis* (Miq.) Kurz exhibited activities combining selective inhibition of pathogenic bacteria and intestinal cancer cells without affecting beneficial bacteria and normal intestinal cells. To the best of our knowledge, this is the first study on antibacterial and antiproliferative activities of *A. polymorphum* Spire, *B. vitis-idaea* (Burm.f.) C.E.C.Fisch., *I. nigricans* R.Br. ex Wight and Arn., *L. cochinchinensis* Pierre ex Gagnep., *P. siamensis* (Miq.) Kurz, and *M. saigonense* (Kuntze) Merr. Moreover, there are no previous studies on the cytotoxic effects of *A. blancoi* (Elmer) Merr. Although the cytotoxic effect of products isolated from *B. malabarica* Roxb. was described previously (Kittakoop et al., 2000), its antibacterial activity is herein reported for the first time. Our results correspond with those of previous studies on antibacterial and antiproliferative activities of *A. grandis* Benth. (Bradacs et al., 2009), *D. esculentum* (Retz.) Sw. (Mackeen et al., 1997; Rahmat et al., 2003), and *P. javanica* Blume (Khan et al., 2001; Win et al., 2015). The above highlighted seven plant extracts with promising activities have mainly been discussed.

Two of the four tested species of the genus *Artocarpus* (Moraceae), namely, *A. blancoi* (Elmer) Merr. and *A. camansi* Blanco, exhibited strong antibacterial and antiproliferative activities in our study. *Artocarpus* spp. are rich in phenolic compounds, such as flavonoids, stilbenoids, and arylbenzofurans, which are known to possess a wide range of biological activities, including antibacterial and anticancer effects (Hafid et al., 2017). Our study is the first to report on anticlostridial activities of *Artocarpus* spp. As flavonoids have been reported to have potent *in vitro* inhibitory effect on some clostridia (Wu et al., 2013), these compounds might be responsible for significant antibacterial activities revealed by *A. blancoi* (Elmer) Merr. and *A. camansi* Blanco against *C. difficile* and *C. perfringens*. Beloy et al. (1976) isolated the flavonoid 5,7,4′-trihydroxyflavanone-3-O-α-L-rhamnopyranoside from the bark extract of *A. blancoi* (Elmer) Merr., showing antibacterial activity against *Mycobacterium tuberculosis*. Ante et al. (2016) showed that bark essential oil of *A. camansi* Blanco produced antibacterial activity against some diarrheagenic bacteria. Our results show that both of these plants inhibited gram-positive bacteria only. Beside their anticlostridial effect, this selectivity probably also contributed to their relative toxicity to beneficial bacteria. *In vitro* inhibitory effect against lactobacilli was previously reported for *Artocarpus lacucha* Buch.-Ham. (Teanpaisan et al., 2014). An example of a compound isolated from the plant of this genus and showing similar activities is artocarpin. In the study by Sato et al. (1996), this flavonoid exhibited strong inhibition of all gram-positive bacteria, including *L. casei*, whereas in another study, it produced higher MICs against *E. coli* and *Pseudomonas aeruginosa* (Septama and Panichayupakaranant, 2015). The absence of antibacterial action of *A. odoratissimus* Blanco found herein will correlate with rather low levels of phenolic content detected in its fruit methanolic extract (Abu Bakar et al., 2015), compared to antibacterially active species (Jalal et al., 2015). Although hexane bark extract of *A. elasticus* Reinw. ex Blume exhibited activity against *B. cereus* and *E. coli* in the study by Ramli et al. (2016), its lack of activity in the present study could be influenced by the use of different extraction procedures. According to our results, *A. blancoi* (Elmer) Merr. and *A. camansi* Blanco had a selective cytotoxic effect on intestinal cancer cells, whereas the former did not show cytotoxicity to normal cells at the inhibitory concentrations against several pathogens. Various terpenoids and phytosterols were previously isolated from methanolic and dichloromethane extract of stem and leaves of *A. camansi* Blanco, respectively. Among them, friedelinol, cycloartenol, and cycloartenol acetate inhibited the growth of HT-29 cells; squalene has profound chemopreventive activity against colon carcinogenesis; and *ß*-sitosterol has been shown to induce apoptosis in human colon tumors (Tsai et al., 2013). Regarding cytotoxic compounds isolated from other *Artocarpus* spp., the prenylated flavone artelastin revealed strong *in vitro* activity against five colon cancer cell lines (COLO 205, HCT 116, HCT 15, HT-29, and SW 620) in the study by Pedro et al. (2005).

Similar to *Artocarpus* spp., the leaf extract of *A. tectorius* (Lour.) Merr. exhibited growth-inhibitory effects only against gram-positive bacteria. This corresponds with the study by Wiart et al. (2004), where its methanolic leaf extract produced antibacterial activity against *B. cereus* but not *E. coli*. We also found that the overall cytotoxic effect of this plant was strong. Although the antiproliferative effect on cancer cells was not selective, the extract concentrations inhibiting the pathogens were generally nontoxic to normal intestinal cells. Previous phytochemical analysis of leaf ethanolic/methanolic extracts of this plant showed the presence of various naphthylisoquinoline alkaloids, such as 7-epiancistrobrevine D, ancistrocladinine, ancistrotectoquinone A-B, ancistrotectoriline A–C, and hamatinine (Anh et al., 1997; Tang et al., 2000; Tang et al., 2010; Bringmann et al., 2016). Since these isoquinoline alkaloids are known to possess various biological activities, including antimicrobial and cytotoxic effects, we suspect them to be responsible for the growth-inhibitory effects revealed by *A. tectorius* (Lour.) Merr. in the present study. For example, in the study by Mihalyi et al. (2014), michellamine B isolated from *Ancistrocladus korupensis* D.W.Thomas and Gereau inhibited *B. subtilis*. Jiang et al. (2013) showed that naphthylisoquinolines isolated from *A. tectorius* (Lour.) Merr. exhibited cytotoxic effect against three leukemia cells *in vitro*. In another study, 7-epiancistrobrevine and ancistrotectoriline exhibited activity against pancreatic cancer cells (Shang et al., 2020). The present study is the first to report on *in vitro* selective antiproliferative activity of *A. tectorius* (Lour.) Merr. against intestinal cells.

Regarding *P. siamensis* (Miq.) Kurz, there are no comparable studies dealing with species of the same genus. However, our results showing a noticeable combination of selective antibacterial and cytotoxic effects of its bark extract can be compared to the data available for closely related genus *Shorea* (Dipterocarpaceae). For example, Marandi et al. (2016) showed that bark ethanolic extract from Indian antidiarrheal and antidysenteric medicinal plant *Shorea robusta* Gaertn. exhibited inhibitory action against *B. cereus*, *B. subtilis*, *E. faecalis*, *E. coli*, *S.* Typhi, and *V. cholerae*. Stilbene derivatives isolated from barks of *Shorea* spp. previously showed strong antibacterial effects against some of these strains (Nitta et al., 2002; Sudto et al., 2019). Some polyphenols, such as stilbenes, can inhibit several nonbeneficial bacteria from the human microbiota, with no noticeable effects on the growth of probiotic bacteria (Requena et al., 2010). Therefore, we suggest that some of these agents could also contribute to the selective antibacterial activities of *P. siamensis* (Miq.) Kurz shown in the present study. Regarding cytotoxic effect, oligostilbenoids were usually the constituents derived from *Shorea* spp., with reported antiproliferative action against various cancer cell lines (Rohaiza, 2011; Zawawi et al., 2012; Moriyama et al., 2016). Among them, ampelopsin E exhibited obvious antiproliferative properties on COLO205 and HT-29 cells (Tian et al., 2019), whereas *α*-viniferin showed selective inhibition of colon cancer cells (HCT-116, HT-29, and Caco-2) with twofold lower IC_50_ compared to normal colon cells (CCD-18Co) (Gonzalez-Sarrias et al., 2011). To identify phytochemicals responsible for the *in vitro* selective inhibitory actions shown by *P. siamensis* (Miq.) Kurz in the present study, an accurate chemical analysis of this plant is needed.

Finally, *E. microphylla* Lam., *L. cochinchinensis* Pierre ex Gagnep., and *M. saigonense* (Kuntze) Merr. revealed a strong selective antiproliferative effect against intestinal cancer lines. It has been reported that triterpenes urs-12-en-24-oic acid, 3-oxo-, methyl ester, and *ß*-amyrin are involved in anticancer activities of products derived from leaves of *E. microphylla* Lam. (Rajkumar et al., 2019). Our study corresponds with other studies dealing with these chemicals. For example, in the study by Kuete et al. (2018), *ß*-amyrin produced a selective cytotoxic effect against Caco-2 compared to that on normal cell line HEK293. In another study, extract of *Alstonia macrophylla* Wall. ex G.Don containing *ß*-amyrin produced a selective cytotoxic effect against HT-29 compared to that on normal cell line HDFn (Tan et al., 2019). The present study is the first to report the antiproliferative activities of *E. microphylla* Lam. against intestinal cell lines. Compounds such as triterpenes, tannins, ellagic acids, glycosides, and flavones were previously attributed to bioactive properties of *Lagerstroemia* spp. (Chan et al., 2014). In previous studies, triterpenes isolated from species of this genus produced significant *in vitro* activity against colon cancer cells, for instance, betulinic acid and 3*β*-acetoxyolean-12-en-28-acid against HCT15 (Woo et al., 2016) and corosolic acid against HCT116 (Sung et al., 2014). Regarding *M. saigonense* (Kuntze) Merr., there are various previously published studies on related species showing corresponding results. For example, the methanolic leaf extract of *Melastoma malabathricum* L. produced an antiproliferative effect on HT-29 in the study by Kamsani et al. (2019). Asiatic acid, caffeic acid, *p*-coumaric acid, kaempferol, quercetin, rutin, and ursolic acid were isolated compounds with previously profound antiproliferative action to this cell line. In the study by Karakurt et al. (2020), *p*-coumaric acid exhibited selective inhibition of Caco-2 and HT-29 cells compared to that of healthy colon epithelial cells (CCD-18Co). Since the decoction from the leaves of *M. malabathricum* L. is also traditionally consumed to treat diarrhea, we suggest a similar composition of bioactive compounds to be present in *M. saigonense* (Kuntze) Merr. (Ong and Nordiana, 1999). Regarding the moderate selective antiproliferative activities of bark and leaf with flower bud extracts of *M. dodecandrum* Lour., three pentacyclic triterpenoids (ursolic acid, asiatic acid, and terminolic acid) and one tannin (casuarinin) were previously isolated from this plant and found to significantly decrease interleukin-8 production in HT-29 (Yang et al., 2014).

In summary, *A. blancoi* (Elmer) Merr., *A. tectorius* (Lour.) Merr., and *P. siamensis* (Miq.) Kurz produced significant growth-inhibitory effects against diarrheagenic bacterial pathogens at concentrations nontoxic to normal intestinal cells. Except the strong anticlostridial actions of *A. blancoi* (Elmer) Merr., the MICs determined for these plant extracts in the present study reflect rather moderate antibacterial activities. However, the discrimination of specific cell toxicity indicates that higher amounts of these products necessary to acquire the appropriate efficiency may still be safe to use (Cos et al., 2006). A long tradition of their use in folk medicinal systems supports this assumption. Moreover, it has been reported that microorganisms are less likely to develop resistance to phytochemicals with anti-infective potential, mainly because of their high diversity in plants. Some were even considered as antibiotic resistance modifying compounds (Sibanda and Okoh, 2007). Additionally, our study showed that the extract of *P. siamensis* (Miq.) Kurz was relatively safe for probiotic bacteria, and together with *A. blancoi* (Elmer) Merr.*,* they exerted selective anticancer activities *in vitro*. Similar to the cytotoxic activities revealed by *E. microphylla* Lam., *L. cochinchinensis* Pierre ex Gagnep., and *M. saigonense* (Kuntze) Merr., the inhibitory effect of *A. blancoi* (Elmer) Merr. on cancer cell line Caco-2 and the selectivity of its overall antiproliferative actions were generally higher than those of anticancer drug 5-fluorouracil.

These results suggest that extracts from the above-mentioned Cambodian and Philippine plant species are promising materials for further research focused on the development of new plant-derived selective antibacterial and antiproliferative agents used in the treatment of infectious diarrhea and associated intestinal cancer diseases. For instance, the combination of strong anticlostridial and anticancer actions of *A. blancoi* (Elmer) Merr. may in the future be utilized in the treatment of digestive cancers associated with *C. difficile* infections (Han et al., 2013). However, further phytochemical and pharmacological research is needed for the isolation and proper identification of their bioactive constituents. Referring to studies dealing with taxonomically related plants to estimate the presence of their bioactive principles is a very limited approach as their composition can vary greatly. On the other hand, our results could serve as an indicator of bioactive potentials of products derived from species of the same taxa. This is mainly the case of *P. siamensis* (Miq.) Kurz that exhibited selective inhibition of pathogenic bacteria and intestinal cancer cells without affecting beneficial bacteria and normal intestinal cells. Future research combining the ethnomedicinal and chemotaxonomic approaches might help to identify more plants with promising bioactivities (Hao and Xiao, 2020).

## Data Availability

The original contributions presented in the study are included in the article/Supplementary Material; further inquiries can be directed to the corresponding author.
